# MiR-324-5p Suppresses Hepatocellular Carcinoma Cell Invasion by Counteracting ECM Degradation through Post-Transcriptionally Downregulating ETS1 and SP1

**DOI:** 10.1371/journal.pone.0133074

**Published:** 2015-07-15

**Authors:** Liangqi Cao, Binhui Xie, Xuewei Yang, Huihong Liang, Xiaofeng Jiang, Dawei Zhang, Ping Xue, De Chen, Zili Shao

**Affiliations:** 1 Department of Hepatobiliary Surgery, the Second Affiliated Hospital of Guangzhou Medical University, Guangzhou, China; 2 Department of General Surgery, the First Affiliated Hospital of Gannan Medical University, Ganzhou, China; Stony Brook University, UNITED STATES

## Abstract

Hepatocellular carcinoma (HCC) is one of the common malignancies, which is highly metastatic and the third common cause of cancer deaths in the world. The invasion and metastasis of cancer cells is a multistep and complex process which is mainly initiated by extracellular matrix (ECM) degradation. Aberrant expression of microRNA has been investigated in HCC and shown to play essential roles during HCC progression. In the present study, we found that microRNA-324-5p (miR-324-5p) was downregulated in both HCC cell lines and tissues. Ectopic miR-324-5p led to the reduction of HCC cells invasive and metastatic capacity, whereas inhibition of miR-324-5p promoted the invasion of HCC cells. Matrix metalloproteinase 2 (MMP2) and MMP9, the major regulators of ECM degradation, were found to be downregulated by ectopic miR-324-5p, while upregulated by miR-324-5p inhibitor. E26 transformation-specific 1 (ETS1) and Specificity protein 1 (SP1), both of which could modulate MMP2 and MMP9 expression and activity, were presented as the direct targets of and downregulated by miR-324-5p. Downregulation of ETS1 and SP1 mediated the inhibitory function of miR-324-5p on HCC migration and invasion. Our study demonstrates that miR-324-5p suppresses hepatocellular carcinoma cell invasion and might provide new clues to invasive HCC therapy.

## Introduction

Hepatocellular carcinoma (HCC) is one of the most frequently occurring malignancies which is highly metastatic and the third most common cause of cancer deaths in the world [1,2]. The incidence rate of HCC in Asian countries is increasing, since chronic hepatitis or liver diseases are prevalent in these areas [1–3]. The prognosis of most patients with HCC is generally very poor, with a survival rates 20–65% for 1 year, 10–30% for 3 years and 10–20% for 5 years. The prognosis of HCC has not been improved despite significant progress in the diagnostic and therapy of HCC. Tumor characteristics including the information of tumor size and number, tumor cell differentiation, and vascular invasion might contribute to HCC patients’ poor prognosis and outcome [4]. Vascular invasion has been reported as the most important and independent predictor of HCC patients’ survival, and has been shown to increase the risk of tumor recurrence after liver transplantation [4,5]. As the invasive ability of HCC cells is instructive to tumor dissemination, it prompts investigators to explore the mechanism underlying HCC invasion involved in tumor recurrence and progression.

The invasion and metastasis of cancer cells is a multistep and complex process which is mainly initiated by extracellular matrix (ECM) degradation [6,7]. The invasive capacity of tumor cells depend on their ability to degrade the protein components of the ECM and basement membranes. Matrix metalloproteinases (MMPs), a family of related zinc-dependent proteinases, could cleave almost any component of the ECM and basement membranes, thereby leading to cancer cells to penetrate and infiltrate the subjacent stromal matrix [8,9]. Over 20 human MMP members have been identified to play essential roles in carcinogenesis, influencing cell adhesion, migration, EMT, and angiogenesis [10]. High level expression or activation of MMPs is supposed to be associated with the metastasis of tumor cells [11,12]. Among all members of MMPs family, MMP-2 and MMP-9 are the most concerned and their functions have been well-characterized during tumor invasive process [13–19]. High expression of MMP-2 and MMP-9 was found to be associated with time to tumor recurrence in HCC patients after surgical resection, while decrease the expression level of MMP-2 and MMP-9 resulted in the inhibition of invasion and metastatic capabilities of hepatocellular carcinoma cells [20–23].

MicroRNAs (miRNAs), a class of endogenous non-coding small RNAs of 20–22 nucleotides, are evolutionarily conserved non-coding RNA molecules. As a new type of gene expression regulators, miRNAs are being found to negatively modulate gene expression by targeting the 3' untranslated region (3'-UTR) of mRNAs in a sequence-specific manner, and are involved in various biological processes [24–26]. Accumulating evidence suggests that miRNAs have key roles in the progression of various human cancers, including cell differentiation, proliferation, apoptosis, metastasis and angiogenesis[27–29]. Aberrant expression of miRNA has been investigated in HCC and shown to play essential roles during HCC progression [30–33].

Herein, the present study found that miR-324-5p was downregulated in both HCC cell lines and tissues. Ectopic miR-324-5p led to the reduction of HCC cells invasive and metastatic capacity, whereas inhibition of miR-324-5p promoted the invasion of HCC cells. Consistently, MMP2 and MMP9, the major regulators of ECM degradation, were downregulated by ectopic miR-324-5p, while upregulated by miR-324-5p inhibitor. Furthermore, E26 transformation-specific 1 (ETS1) and Specificity protein 1 (SP1), both of which could modulate MMP2 and MMP9 expression and activity, were presented as the direct targets of and downregulated by miR-324-5p. Downregulation of ETS1 and SP1 mediated the inhibitory function of miR-324-5p on HCC migration and invasion. In conclusion, our study demonstrates that miR-324-5p suppresses hepatocellular carcinoma cell invasion by attenuate the expression and activity of MMP2 and MMP9, subsequently counteracting ECM degradation, through post-transcriptionally downregulating ETS1 and SP1.

## Material and Methods

### Cell culture

The immortalized normal liver epithelial cells, THLE-3, were purchased from the American Type Culture Collection (ATCC, Manassas, VA, USA) and were cultured under the conditions stated by the manufacturer. The HCC cell lines: HepG2, Hep3B, MHCC97H, MHCC97L, BEL-7402, Huh7, SMMC-7721, PLC/PRF/5, and QGY-7703 were kindly donated by Prof. Qian Wang (Sun Yat-Sen University, Guangzhou, China), which HepG2, Hep3B and PLC/PRF/5 were purchased from ATCC, and MHCC97H, MHCC97L, BEL-7402, Huh7, SMMC-7721 and QGY-7703 were purchased from Shanghai Yansheng industrial Co. (Shanghai, China). The HCC cells were grown in Dulbecco’s modified Eagle’s medium (DMEM, Gibco-BRL, Grand Island, NY, USA) supplemented with 10% fetal bovine serum (FBS, Gibco-BRL) supplemented with 100 U/ml penicillin and 100 μg/ml streptomycin (Gibco-BRL), at 37°C in a 5% CO_2_ atmosphere in a humidified incubator.

### Tissue specimens

Eleven pairs of snap-frozen HCC tumor and matched adjacent non-tumor tissues were diagnosed histopathologically at the Second Affiliated Hospital of Guangzhou Medical University from January to December in 2013. The tissues were immediately stored in liquid nitrogen after surgery until further use. For the use of the clinical materials for research purposes, the Institutional Research Ethics Committee approved the study, and prior patient consent was obtained.

### Ethics statement

For the use of clinical materials for research purposes, written informed consent was obtained from all participating patients who were informed of, and understood before tissue collection. This study was approved by the Medical Ethics Committee of the Second Affiliated Hospital of Guangzhou Medical University, Guangzhou, China.

### Generation of stably engineered cell lines

For construction of miRNA expression plasmid, miR-324-5p precursors were amplified from human genomic DNA by PCR using the primer pairs: forward, 5'-CGCGGATCCGGGTGGATGTAAGGGATGAG-3'; reverse, 5'-CCGGAATTCTTGGGCTGATCCAGGAGAAG-3'; PCR products were cloned into the retroviral transfer plasmid pMSCV-puro (Clontech Laboratories Inc., Mountain View, CA, USA). miR-324-5p expression plasmid was then cotransfected with packaging plasmid into 293FT cells, using the standard calcium phosphate transfection method previously reported [34]. Thirty-six hours after cotransfection, supernatants were collected and incubated with HCC cells to be infected for 24 hours in the presence of polybrene (2.5 μg/ml, Sigma, Saint Louis, MO, USA). After infection, puromycin (1.5 μg/ml, Sigma) was used to select stably transduced cells about 14 days. The expression of miR-324-5p was confirmed by real-time quantitative PCR (qRT-PCR).

### miRNAs, small interfering RNA (siRNA) and transfection

A micrON hsa-miR-324-5p mimic (No. miR10000761-1-5), a micrOFF hsa-miR-324-5p inhibitor (No. miR20000761-1-5), and the negative controls [NCs; micrON mimic Negative Control #22 (No. miR01101-1-2) and micrOFF inhibitor Negative Control #22 (No. miR02101-1-2)] were purchased from RiboBio (RiboBio Co. Ltd, Guangzhou, China). For depletion of ETS1 and SP1, the siRNAs were synthesized and purified by RiboBio (The *ETS1* siRNA sequence was GCAAGGACCUAGCAACACUUA; the *SP1* siRNA sequence was CCCAATGGACAGGTCAGTT). Transfection of oligonucleotides and siRNAs were performed using Lipofectamine 2000 (Invitrogen, Carlsbad, CA, USA), according to the manufacturer’s protocol. Total-RNA and protein were extracted 48 h after transfection for use in qRT-PCR and western blot analysis.

### RNA extraction and qRT-PCR

Total-RNA was extracted with TRIzol reagent (Invitrogen) according to the manufacturer’s instructions. cDNA was synthesized using GoTaq 2-Step RT-qPCR System (Promega, Madison, WI, USA) according to the manufacturer’s instructions. qRT-PCR reactions were prepared using SYBR Premix Ex Taq (Takara, Kyoto, Japan). Reactions were performed in triplicate using ABI Prism 7500 Sequence Detection System (Applied Biosystems, Foster City, CA, USA). The PCR primers were: MMP-2, 5'- AGGCCAAGTGGTCCGTGTGA-3'; 5'-TAGGTGGTGGAGCACCA GAG-3'; *MMP9* forward: 5'- ATCCGGCACCTCTATGGTCCTC-3'; *MMP9* reverse: 5'- GCACAGTAGTGGCCGTAGAAGG-3'; *GAPDH* forward: 5'- GACTCATGA CCACAGTCCATGC -3', *GAPDH* reverse: 5'- AGAGGCAGGGAT GATGTTCTG -3'. The qRT-PCR conditions for genes were set at 95°C for 10 min, followed by 40 cycles at 95°C for 20 s, 60°C for 30 s and 72°C for 1 min.The qRT-PCR reactions for miRNAs were performed at 95°C for 3 min, followed by 40 cycles (each 30 s in length) at 95°C, 58°C, and 72°C. Expression levels of genes were normalized to that of the housekeeping gene *GAPDH* as the control and calculated as 2^-[(Ct of Gene)–(Ct of GAPDH)]^; the relative expression of the miRNA were calculated as 2^-[(Ct of miRNA)–(Ct of U6)]^ after normalization with reference to the expression of small nuclear RNA U6. MiR-103 was chosen as the negative control microRNA according to previous report [35]. C_t_ represents the threshold cycle for each transcript.

### Western blot analysis

Total cell lysates were prepared in 1× sodium dodecyl sulfate (SDS) buffer (50 mM Tris-Cl (pH 6.8), 100 mM DTT, 2% SDS, 0.1% bromophenol blue, 10% glycerol). Equal quantities of protein were electrophoresed through SDS-PAGE and transferred onto polyvinylidene fluoride (PVDF) membranes (Millipore, Billerica, MA, USA). Membranes were probed with antibodies to ETS1, SP1, MMP2, MMP9 and β-actin (#6258; #5931; #13132; #3852; #4967; Cell Signaling Technology, Danvers, MA, USA) MMP1 and MMP3 (SAB1406131; SAB1406132; Sigma), respectively

### Transwell matrix penetration assay

For migration and invasion assays, cells (2×10^4^) to be tested were plated on the top side of the polycarbonate Transwell filter with Matrigel (BD Biosciences, San Jose, CA) coating in the upper chamber of the BioCoat Invasion Chambers (BD, Bedford, MA, USA) and incubated at 37°C for 24 hrs, followed by removal of cells inside the upper chamber with cotton swabs. Invaded cells on the membrane bottom-surface were fixed in 1% paraformaldehyde, stained with 0.2% (w/v) crystal violet solution for 15 min, and Cells adhering to the undersurface of the filter were counted (Ten random 100× fields per well) using an inverted microscope. Three independent experiments were performed and the data are presented as mean ± standard deviation (SD).

### Wound healing assay

Cell migrating ability was measured using the scratch assay. Briefly, cells were seeded on six-well plates with DMEM containing 10% FBS and grown to monolayer confluency. The cell monolayers were scratched with a sterile pipette tip to create straight wounds. At 0 and 24 hr after wounding, respectively, migration images were captured and documented at different time points using an inverted Olympus IX50 microscope with 10× objective lens and the Image-Pro Plus software (Media Cybernetics).

### 3-Dimensional Cell Culture

The Matrigel matrix (BD Biosciences) was used in 3-dimensional cell culture, which displays morphologies typical of highly aggressive invasiveness presenting more outward projections (Invadopodia or invasive feet). Cells (1×10^4^) were trypsinized and seeded in 24-well plates coated with Matrigel (BD Biosciences). Pictures were taken under microscope at every 2 days.

### Enzyme-linked immunosorbent assay (ELISA)

For determination of the secretion of MMP2 and MMP9, human MMP2 (ab100606) and MMP9 ELISA kit (ab100610) from Abcam (Cambridge, UK) were used. The culture medium from each well was collected and tested according to previous study [36,37]. Briefly, 100μl of diluted standard and tested samples, were added to the plate and incubated at 36°C for 90min. After the unbound samples were washed off by DI water and PBS-Triton, specific antibody was incubated with the plate at 36°C for 60min. 100μl of second antibody was added and incubated for another 60min. The substrate was then added and incubated for 60min before the reaction was stopped, followed by the results-reading with a microplate reader. Colorimetric measurement was recorded as OD450 readings. The relative MMP amounts in the indicated cells were expressed as the percentages of those in the DMSO-treated control cells.

### Luciferase reporter assays

For the construction of luciferase reporter vectors, 3′UTR segments of *ETS1 and SP1* were amplified from human genomic DNA using the primer pairs: *ETS1*-3′UTR forward, 5′- CGGGGTACCTGAGTTGTGGACCCATTAGC-3′; reverse, 5′- CCGCTCGAGCCACCTTAGAGCTTAAGACG -3′; *SP1*-3′UTR forward, 5′- CGGGGTACCAGAGGGCATTGCCCGTCTTG -3′ and reverse, 5′- CCGCTCGAGCCTTCAAAGAGGCACTGATG -3. Mutant inserts containing substitutions in the miRNA complementary sites were generated by PCR using the primers: *ETS1*-3′UTR-mut forward, 5′- TGGGATGAAACATGTTTTGGGGGGGGC CTCACTGAAAATCTGAGAA CTATTTACC-3′ and reverse, 5′- GGTAAATAGTT CTCAGATTTTC AGTGAGGCCCCCCCCAAAACATGTTTCATCCCA -3′; *SP1*-3′UTR-mut forward, 5′- ATATGGGCCATACCCCTTAACCCCGGG CCTCAAGGTAGC ATGGGTCCAAGAGACAT -3′ and reverse, 5′- ATGTC TCTTGGACCCA TGCTACCTTGAGGCCCGGGGTTAAGGGGTATG GCCCATAT -3′. PCR products were cloned into the modified pGL3 control vector (Promega, Madison, WI, USA) immediately downstream of the stop codon of the luciferase gene. Wild-type and mutant inserts were confirmed by sequencing. Cells were seeded in triplicate in 24-well plate to reach 80–90% confluency. pGL3- *ETS1* or *SP1*-3′UTR-luciferase plasmid, or mutant plasmid (100 ng) was transfected into HCC cells using the Lipofectamine 2000 reagent, according to the manufacturer’s instruction. Luciferase activity was measured by Dual Luciferase Reporter Assay Kit (Promega) 48 h after transfection. Three independent experiments were performed and the data were presented as the mean ± SD.

### Statistical analysis

All data were expressed as the mean ± SD. The two-tailed Student’s *t* test was used to evaluate the significance of the differences between two groups of data in all experiments. A value of *P* < 0.05 was considered statistically significant.

## Results

### MiR-324-5p is downregulated in both HCC cell lines and tissues

**Fig 1:**
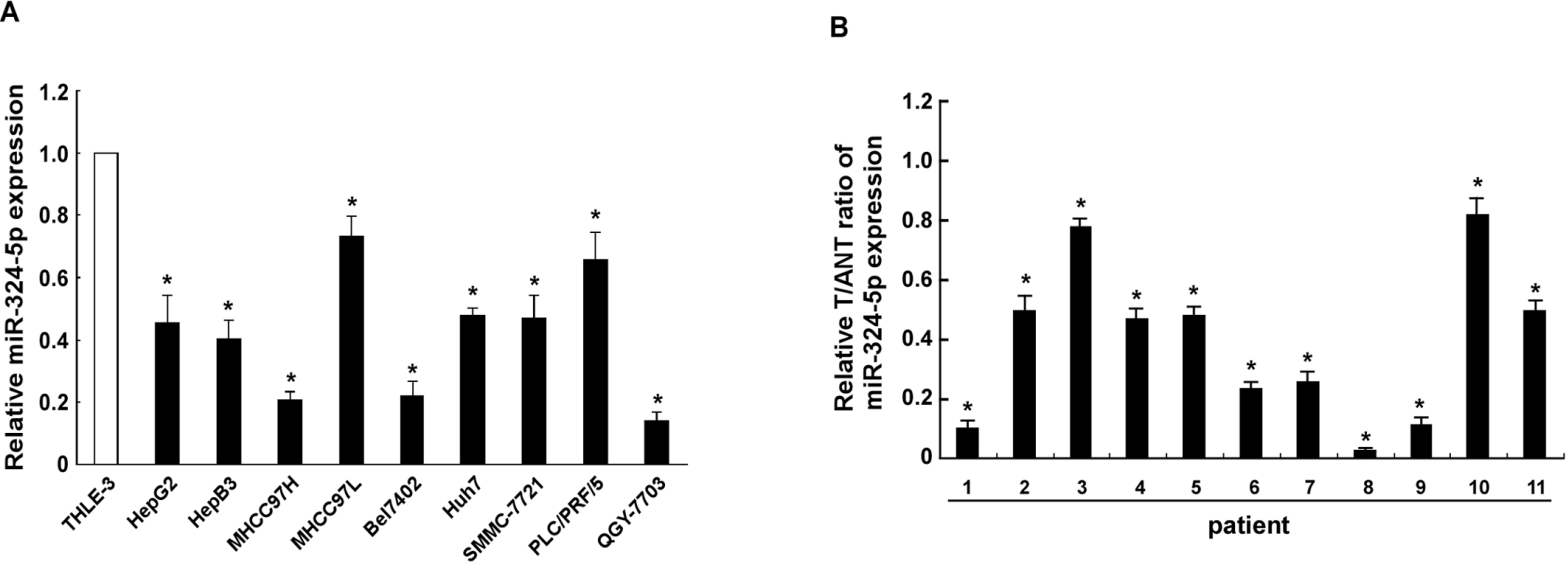
MiR-324-5p is downregulated in both HCC cell lines and tissues. **A.** Real-time PCR analysis of miR-324-5p expression in hepatocellular carcinoma cell lines (HepG2, Hep3B, MHCC97H, MHCC97L, BEL-7402, Huh7, SMMC-7721, PLC/PRF/5 and QGY-7703), compared with normal liver epithelial THLE3 cells. **B.** The expression of miR-324-5p was examined in eleven paired cancerous tissues (T) and their adjacent noncancerous hepatic tissues (ANT). The result is performed as the ratio of T and ANT. The average miR-324-5p expression was normalized using U6 expression. Each bar represents the mean ± SD of three independent experiments. * *P* <0.05.

To determine which miRNA species may be involved in HCC progression, we first compared miRNA expression profiles in matched pairs of HCC and adjacent non-tumor hepatic tissues from 4 patients. The results showed miR-324-5P was significantly downregulated among series of dysregulated miRNAs (S1 Fig). To further confirm the expression of miR-324-5p in HCC, real-time PCR analysis was used. The results showed that miR-324-5p expression, not control miRNA-103, was significantly decreased in all nine HCC cell lines, including HepG2, Hep3B, MHCC97H, MHCC97L, BEL-7402, Huh7, SMMC-7721, PLC/PRF/5 and QGY-7703, compared with that in the immortalized normal liver epithelial cells THLE3 (Fig 1A; S2A Fig). Comparative analysis also revealed that the expression of miR-324-5p, not control miRNA-103, was markedly downregulated in eleven pairs of cancerous tissues compared with the adjacent noncancerous hepatic tissues (Fig 1B; S2B Fig). Taken together, all these data suggested that the expression of miR-324-5p decreases in both HCC cell lines and tissues.

### Ectopic expression of miR-324-5p inhibits HCC cells migration and invasion

**Fig 2:**
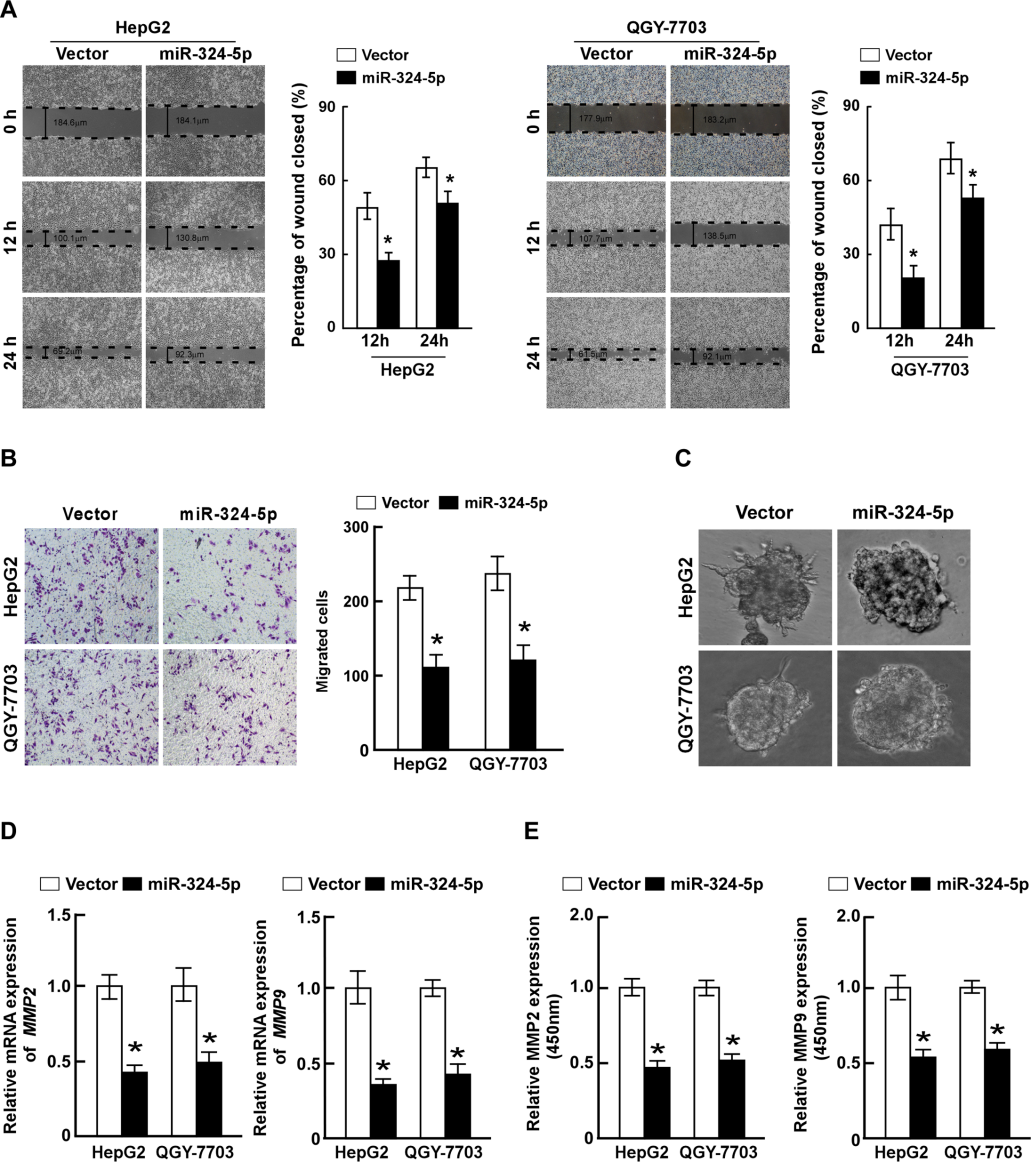
Ectopic expression of miR-324-5p inhibits HCC cells migration and invasion. **A.** Representative micrographs (left) and quantifications (right) of wound healing assay of the indicated cells. Wound closures were photographed at 0, 12 and 24 hours after wounding. **B.** Representative micrographs (left) and quantifications (right) of indicated invading cells in a Matrigel-coated Transwell assay. **C.** Representative micrographs of indicated cells grown on Matrigel for 10 days in 3-Dimensional Cell Culture. **D.** Real-time PCR analysis of MMP2 and MMP9 expression in indicated cells. *GAPDH* served as control. **E.** The activity of MMP2 and MMP9 in indicated cells determined by ELISA assay. Each bar represents the mean ± SD of three independent experiments. * *P* <0.05.

To determine the bio-function of miR-324-5p on HCC progression, HepG2 and QGY-7703 cells stably overexpressing miR-324-5p were established for the following study (S3 Fig). Wound healing assays demonstrated that ectopic miR-324-5p attenuated the migration of HCC cells (Fig 2A). Transwell matrix penetration assay revealed that ectopic miR-324-5p significantly decreased the migratory and invasive ability of both HCC cell lines, as compared with that of control cells (Fig 2B). Furthermore, the 3-dimensional cell culture assay demonstrated that miR-324-5p-overexpressing cells displayed cellular morphologies typical of the less invasive phenotype, as the cells presented immotile and spheroid morphology, compared to cells in the control group (Fig 2C). Since MMP2 and MMP9, which were suggested to play essential functions on ECM degradation and promote invasiveness of cancer cells, the expressions and activities of MMP2 and MMP9 were determined by qRT-PCR and ELISA assay. The results showed both the expression and activity of MMP2 or MMP9 mRNA were downregulated by ectopic miR-324-5p (Fig 2D and 2E). Collectively, these data revealed that overexpression of miR-324-5p inhibited the migration and invasiveness of HCC cells.

### Inhibition of miR-324-5p promotes invasion and migration of HCC cells

**Fig 3:**
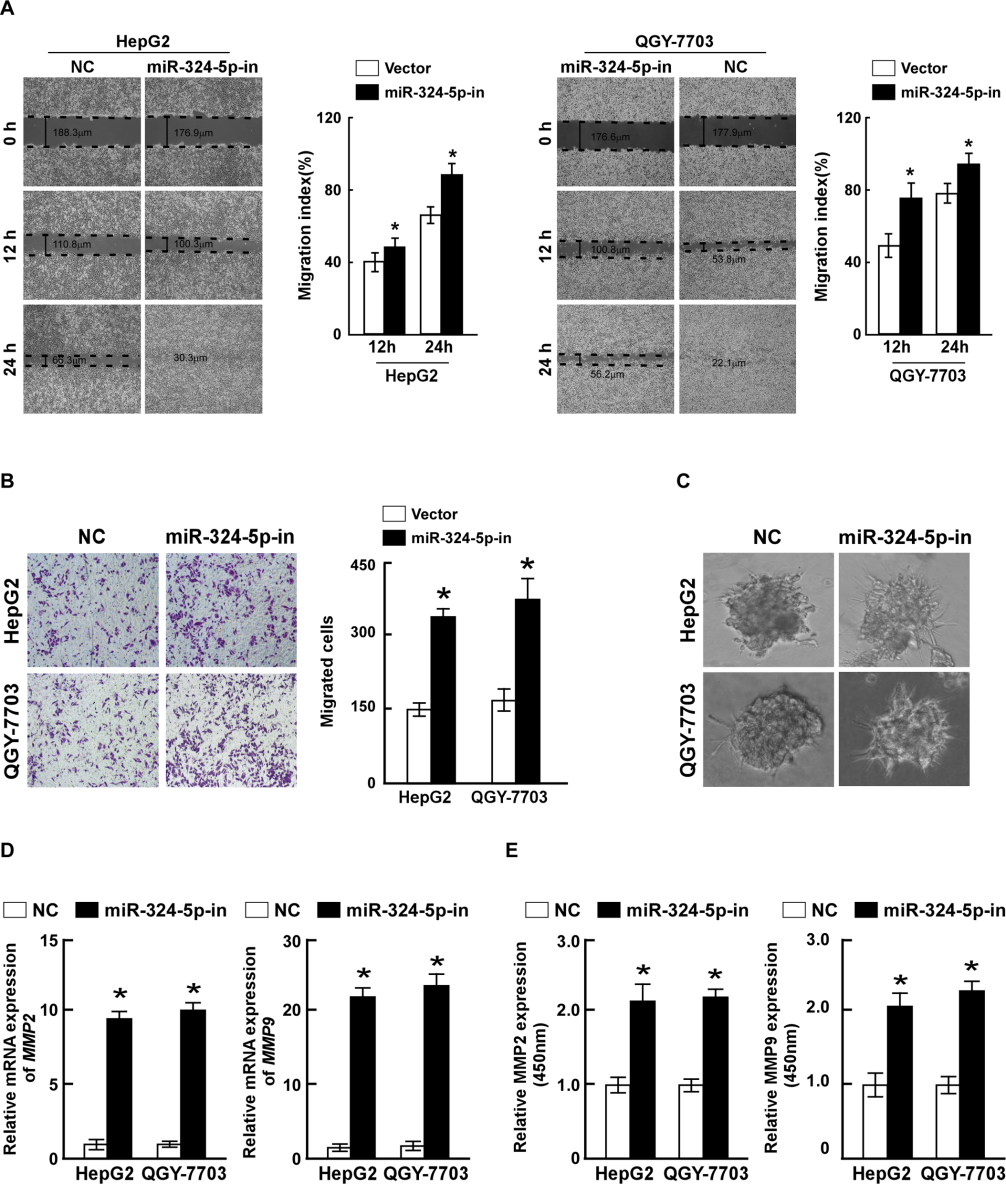
Inhibition of miR-324-5p promotes invasion and migration of HCC cells. **A.** Representative micrographs (left) and quantifications (right) of wound healing assay of the HCC cells, transfected with miR-324-5p inhibitor or negative control (NC). Wound closures were photographed at 0, 12 and 24 hours after wounding. **B.** Representative micrographs (left) and quantifications (right) of indicated invading cells in a Matrigel-coated Transwell assay. **C.** Representative micrographs of indicated cells grown on Matrigel for 10 days in 3-Dimensional Cell Culture. **D.** Real-time PCR analysis of MMP2 and MMP9 expression in indicated cells. *GAPDH* served as control. **E.** The activity of MMP2 and MMP9 in indicated cells determined by ELISA assay. Each bar represents the mean ± SD of three independent experiments. * *P* <0.05.

To further test whether endogenous miR-324-5p contributes to the inhibition of HCC cells migration and invasion, loss-of-function studies using a miR-324-5p inhibitor were represented. Inhibition of miR-324-5p dramatically promoted the migration and invasion of both HepG2 and QGY-7703 cells. Wound healing assays revealed that inhibition of miR-324-5p accelerated the migration of HCC cells (Fig 3A). Transwell matrix penetration assay showed that inhibition of miR-324-5p increased the migratory and invasive ability of both HCC cells (Fig 3B). 3-dimensional cell culture assay showed that inhibition of miR-324-5p cells displayed cellular morphologies typical of a highly invasive phenotype, as the cells presented much more outward projections, compared with cells in control group (Fig 3C). Consistently, the expressions and activities of MMP2 and MMP9, determined by qRT-PCR and ELISA, were elevated by miR-324-5p inhibitor (Fig 3D and 3E). These results revealed that downregulation of miR-324-5p could enhance the migration and invasion of HCC cells, which further confirmed the bio-function of miR-324-5p on HCC progression.

### ETS1 and SP1 are the direct targets of miR-324-5p

**Fig 4:**
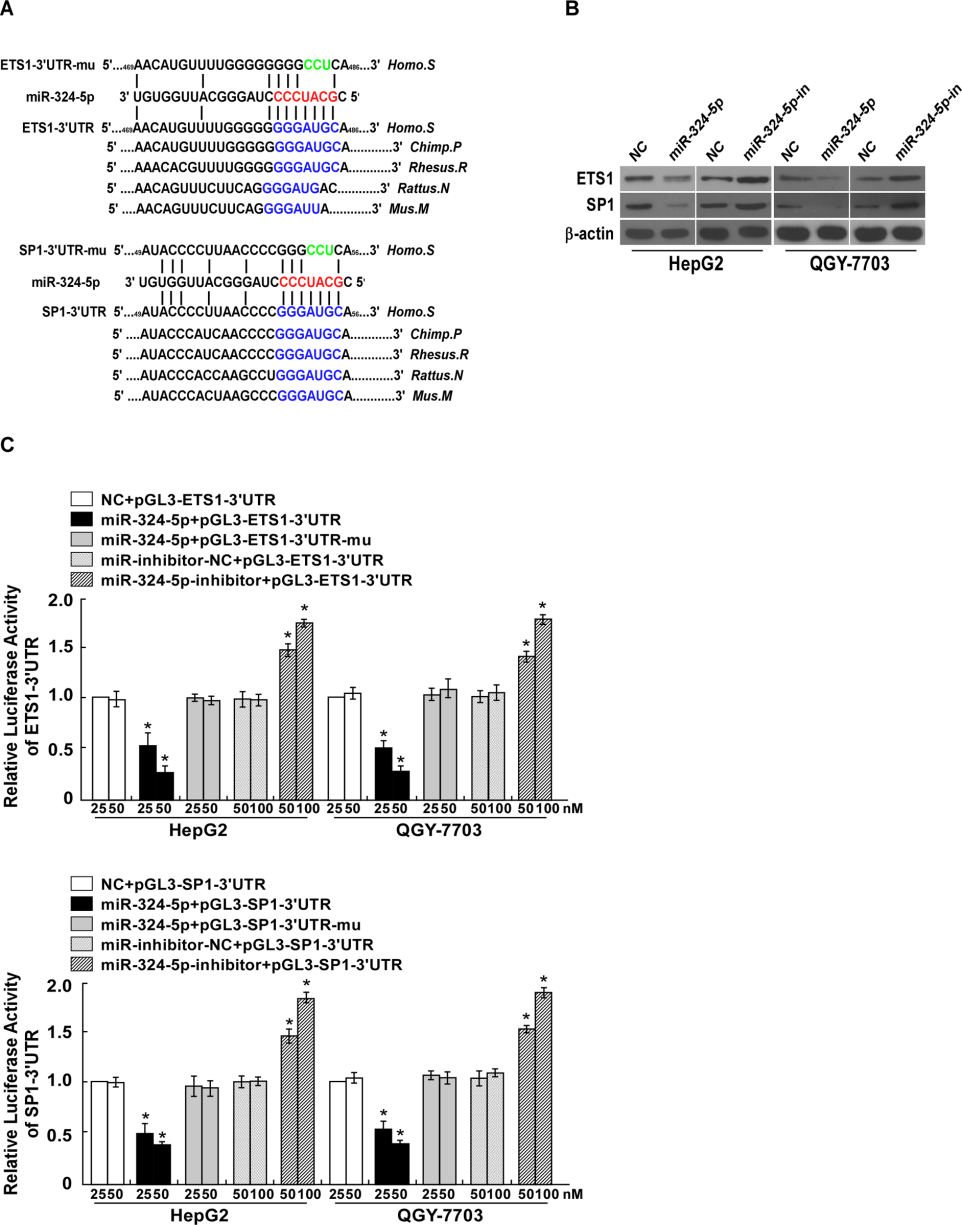
ETS1 and SP1 are the direct targets of miR-324-5p. **A.** Schematic representation of the mature miR-324-5p sequence, miR-324-5p target site in the 3'-UTR of *ETS1* and *SP1* mRNA and a 3'-UTR mutant of *ETS1* and *SP1* mRNA containing three altered nucleotides in the putative target site (shown as *ETS1* or *SP1*-3'UTR-mu). **B.** The expression levels of ETS1 and SP1 protein in HCC cells overexpressing miR-324-5p or transfected with miR-324-5p inhibitor, compared with control cells, by western blotting 48 hours after transfection; α-Tubulin served as the loading control. *C.* Luciferase assay of pGL3- *ETS1*-3'UTR or pGL3- *ETS1*-3'UTR-mut reporter, pGL3- *SP1*-3'UTR or pGL3- *SP1*-3'UTR-mut reporter cotransfected with different amounts (25, 50nM) of miR-324-5p mimic in indicated cells, or different amounts (50, 100nM) of miR-324-5p inhibitor, compared with negative control (NC). Each bar represents the mean ± SD of three independent experiments. * *P* <0.05.

To understand the mechanism by which miR-324-5p suppressed the migration and invasiveness of HCC cells, the publicly available algorithm TargetScan, which is public compilation of database used for microRNAs and their targets, was used to select potential target(s) of miR-324-5p. The results indicated that ETS1 and SP1 were two of the potential targets of miR-324-5p (Fig 4A). Western blotting revealed that the expression of ETS1 and SP1 decreased in HepG2 and QGY-7703 overexpressing miR-324-5p and increased in cells transfected with the miR-324-5p inhibitor (Fig 4B). To further confirm whether ETS1 and SP1 were the direct targets of miR-324-5p, the luciferase reporter of ETS1-3'-UTR and SP1-3'-UTR containing miR-324-5p binding sites, were constructed. The results showed that ectopic expression of miR-324-5p decreased, while inhibition of miR-324-5p increased, the luciferase activity of the ETS1-3'-UTR or SP1-3'-UTR-luciferase reporter. By contrast, the ETS1-3'-UTR or SP1-3'-UTR- luciferase reporter with a mutant miR-324-5p binding site was not inhibited by ectopic miR-324-5p (Fig 4C). Collectively, our results suggested that miR-324-5p directly targets and downregulates ETS1 and SP1.

### ETS1 and SP1 suppression are essential for miR-324-5p-inhibited cell migration, invasion and ECM degradation in HCC

**Fig 5:**
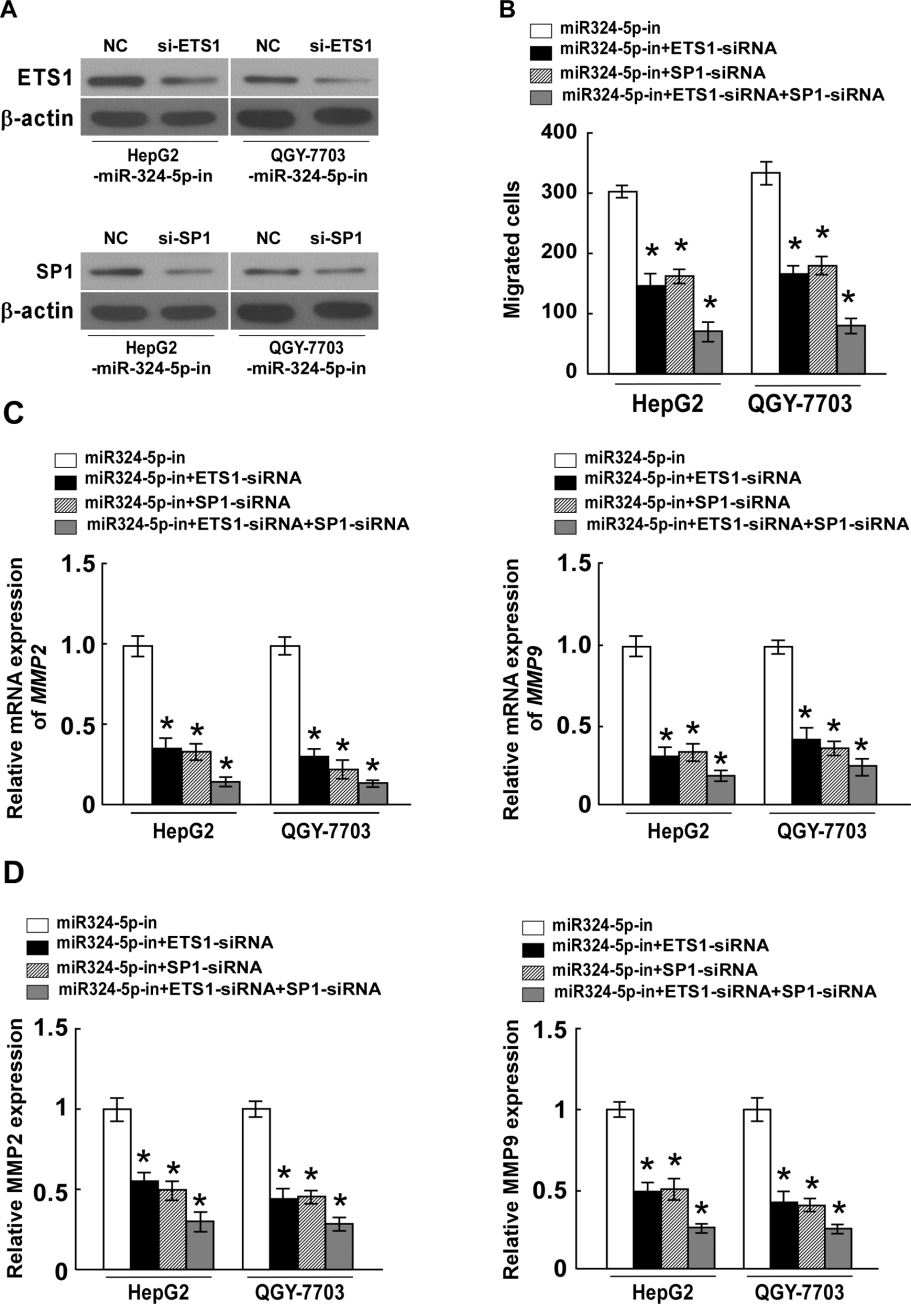
ETS1 and SP1 suppression are essential for miR-324-5p-inhibited cell migration, invasion and ECM degradation in HCC. **A.** The expression levels of ETS1 or SP1 in miR-324-5p-inhibitor transfected HCC cells that were transfected with ETS1 or SP1-siRNA, as measured by western blotting; α-Tubulin served as the loading control. **B.** Quantification of indicated invading cells in a Matrigel-coated Transwell assay. **C.** Real-time PCR analysis of MMP2 and MMP9 expression in indicated cells. *GAPDH* served as control. **D.** The activity of MMP2 and MMP9 in indicated cells determined by ELISA assay. Each bar represents the mean ± SD of three independent experiments. * *P* <0.05.

To examine the effect of ETS1 or SP1 suppression on miR-324-5p-inhibited cell migration, invasion and ECM degradation in HCC, we suppressed endogenous ETS1 or SP1 expression with an ETS1 or SP1-specific siRNA. The expression of ETS1 or SP1 in HCC cells silencing ETS1 or SP1 was confirmed by western blotting (Fig 5A). The invasive ability of miR-324-5p-inhibited HCC cells transfected with ETS1 or SP1-specific siRNA, or both siRNAs, measured by transwell matrix penetration assay, revealed that silencing ETS1 or SP1 expression in miR-324-5p-repressed cells could reverse the promotive effect of the miR-324-5p inhibitor on HCC cell migration and invasion (Fig 5B). In addition, the results showed that the reverse function by siRNA was more obvious in miR-324-5p-repressed cells transfected with both ETS1 and SP1 siRNAs. Consistently, the expressions and activities of MMP2 and MMP9 obtained the similar results, as shown in Fig 5C and 5D, indicating that silencing ETS1 or SP1, or both ETS1 and SP1, reversed the promotive function of miR-324-5p inhibitor on ECM degradation. All of these results further confirmed that ETS1 and SP1 suppression play essential roles in miR-324-5p-inhibited cell migration, invasion and ECM degradation in HCC.

## Discussion

The present study, miR-324-5p was found to be downregulated in HCC cell lines and tissues. Ectopic miR-324-5p could repress the migration and invasion of HCC cells, while inhibition of miR-324-5p enhances the migratory and invasive capacity of HCC cells. Our study further identified that, as bona fide targets of miR-324-5p, ETS1 and SP1 were both downregulated by miR-324-5p in HCC, finally resulting in inhibition of aggressiveness of HCC cells. In addition, MMP2 and MMP9, which are the major regulators of ECM degradation and also the downstream targets of ETS1 and SP1, and, downregulated by ectopic miR-324-5p, might mediate the inhibition function of miR-324-5p on migration and invasion of HCC cells. These findings report, for the first time, the involvement of miR-324-5p in HCC progression and reveal a novel connection between microRNA, ECM degradation and HCC aggressiveness.

ETS1 is one of the members of ETS family and has been reported to promote invasive behavior in multiple tumors [38–41]. ETS1 has been reported to be upregulated in a variety of tumors and could be considered as a prognostic marker in patients with tumors including breast cancer, ovary and cervix carcinoma and hepatocellular carcinoma [42–46]. Moreover, ETS1 expression is increased in invasive higher grade tumors, and correlates with a higher incidence of lymph node metastasis [41,47,48]. SP-1 is a ubiquitously expressed transcription factor, which belongs to the C2H2-type zinc-finger protein family, and is found to be associated with multiple cellular processes, such as cell differentiation, proliferation, apoptosis, especially during cancer progression [49,50]. It was reported that SP1 is upregulated in multiple tumors and correlate with tumor progression [51–54]. It is of great value to explore the mechanism of ETS and SP1 modulation during tumor progression.

MMPs were considered to play an important role in metastatic spread of tumor cells via ECM degradation. The modulation of MMPs has been under multiple investigations. A growing number of evidences showed that ETS1 plays a key role in promotion of invasive capacity in multiple cancers by regulation of MMPs, including MMP-1, MMP-2, MMP-3, MMP-9 and urokinase type plasminogen activator (uPA). ETS1 is found to be co-expressed with MMP-1 and MMP-9 in angiosarcoma of the skin, ovarial carcinoma cells and stromal fibroblasts in breast and ovarian cancer [55–57]. Overexpression of SP1 is found to facilitate MMP-2-mediated cell invasiveness and predicts poor clinical outcome of glioma patients [52]. SP1 is reported as one of the transcription factors that bind to MMP-9 promoter and induces MMP-9 transcription [58]. All of the recent studies revealed that ETS1 and SP1 are responsible for cancer cell invasion via modulation of MMPs. In the present study, we performed data to show that ectopic miR-324-5p inhibited MMP2 and MMP9 expressions and activities through targeting ETS1 and SP1, finally results in suppression of ECM degradation and HCC cell migration and invasion.

However, MMPs are a large family with at least 26 members known and previous studies suggested that other MMP members, such as MMP1 and MMP3, might be regulated by ETS1[55–57]. The expression of MMP1 and MMP3 has been examined in our study and the results showed a barely detectable repression of MMP1 and MMP3 by ectopic miR-324-5p, compared with the repression level of MMP2 and MMP9 (S4 Fig). Moreover, both ETS and SP1 are reported to promote the transcriptional activity of MTA2, which belongs to metastasis associated family and is highly expressed in tumors, resulting in promotion of cancer cell aggressiveness [59,60]. Therefore, although the regulatory function of ETS and SP1 by miR-324-5p on HCC invasion was demonstrated in this study, the in-detailed mechanisms of miR-324-5p in cancer ECM degradation and invasiveness need to be further clarified.

In summary, aberrant expression of miR-324-5p in HCC cells participates in cell mobility and invasion. Overexpression of miR-324-5p could downregualte the expression of ETS and SP1 and might contribute to suppress the ECM degradation by inhibition of MMP2 and MMP9 in HCC. MiR-324-5p might be considered as a novel potential target for invasive HCC treatment.

## Supporting Information

S1 Fig miR-324-5p is downregulated in HCC. miRNA expression profiles in matched pairs of HCC and adjacent non-tumor hepatic tissues from 4 patients.
(TIF)

S2 Fig The expression of miR-103 in both HCC cell lines and tissues. **A.** Real-time PCR analysis of miR-103 expression in hepatocellular carcinoma cell lines (HepG2, Hep3B, MHCC97H, MHCC97L, BEL-7402, Huh7, SMMC-7721, PLC/PRF/5 and QGY-7703), compared with normal liver epithelial THLE3 cells. **B.** The expression of miR-103 was examined in eleven paired cancerous tissues (T) and their adjacent noncancerous hepatic tissues (ANT). The result is performed as the ratio of T and ANT. The average miR-324-5p expression was normalized using U6 expression. Each bar represents the mean ± SD of three independent experiments. * *P*<0.05.
(TIF)

S3 Fig The expression of miR-324-5p in HCC cell lines stably expressing miR-324-5p, determined by Real-time PCR analysis. (TIF)

S4 Fig The expression of MMPs in indicated HCC cells. **A.** The expression levels of MMP1, MMP3, MMP2 and MMP9 protein in HCC cells overexpressing miR-324-5p or transfected with miR-324-5p inhibitor, compared with control cells, by western blotting. GAPDH serves as the loading control. **B.** The mRNA expression levels of MMP1 and MMP3, determined by Real-time PCR analysis. **C.** The activity of MMP1 and MMP3 in indicated cells determined by ELISA assay. Each bar represents the mean ± SD of three independent experiments.
(TIF)
