# Supplementary figures and images for: MiR-324-5p Suppresses Hepatocellular Carcinoma Cell Invasion by Counteracting ECM Degradation through Post-Transcriptionally Downregulating ETS1 and SP1
Source: PLoS One. 2015 Jul 15;10(7):e0133074. doi: 10.1371/journal.pone.0133074 (PMC4503725; doi:10.1371/journal.pone.0133074)

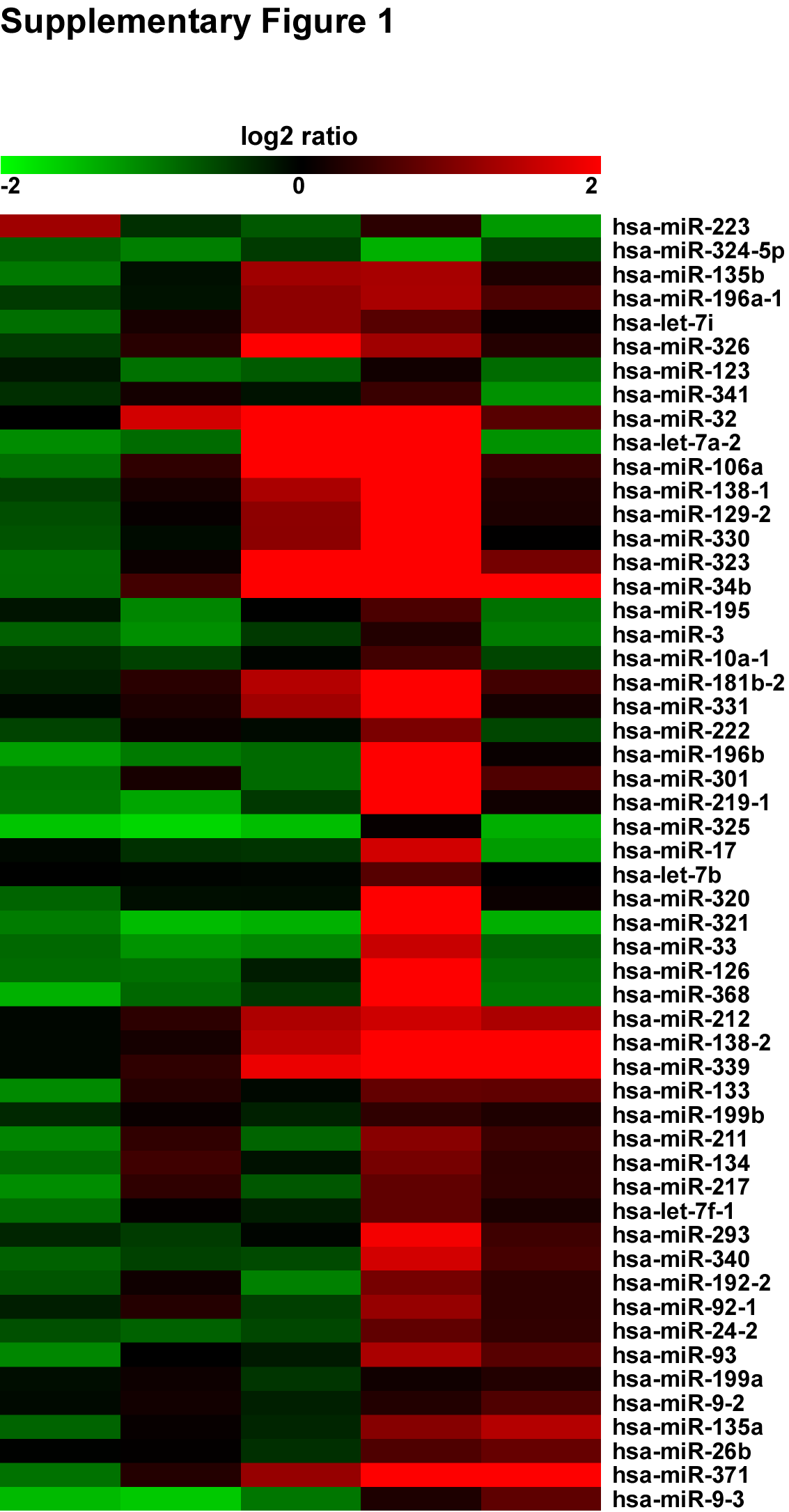

Supplement: S1 Fig — miRNA expression profiles in matched pairs of HCC and adjacent non-tumor hepatic tissues from 4 patients. (TIF) [file pone.0133074.s001.tif]

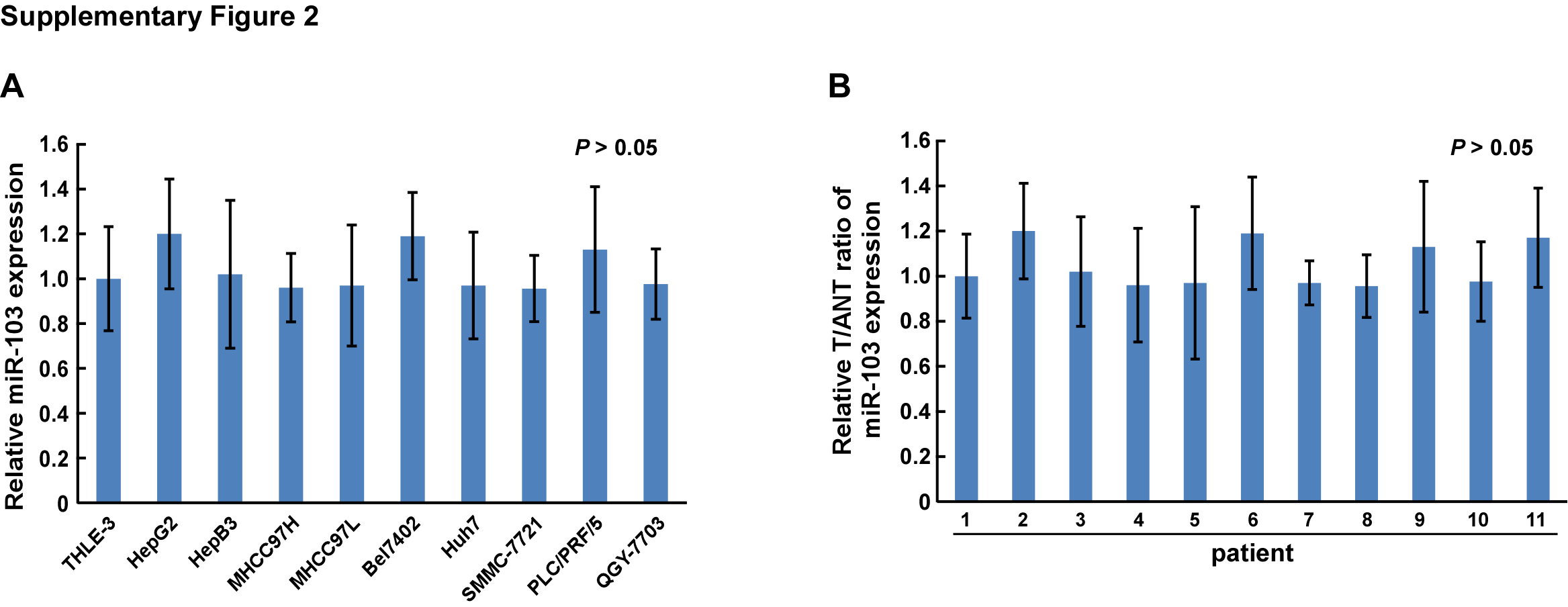

Supplement: S2 Fig — A. Real-time PCR analysis of miR-103 expression in hepatocellular carcinoma cell lines (HepG2, Hep3B, MHCC97H, MHCC97L, BEL-7402, Huh7, SMMC-7721, PLC/PRF/5 and QGY-7703), compared with normal liver epithelial THLE3 cells. B. The expression of miR-103 was examined in eleven paired cancerous tissues (T) and their adjacent noncancerous hepatic tissues (ANT). The result is performed as the ratio of T and ANT. The average miR-324-5p expression was normalized using U6 expression. Each bar represents the mean ± SD of three independent experiments. * P<0.05. (TIF) [file pone.0133074.s002.tif]

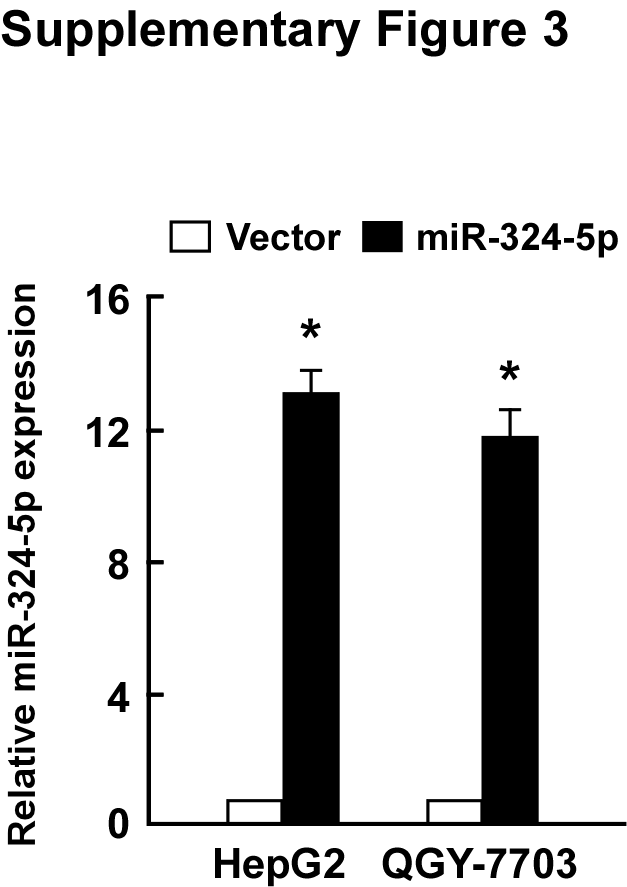

Supplement: S3 Fig — (TIF) [file pone.0133074.s003.tif]

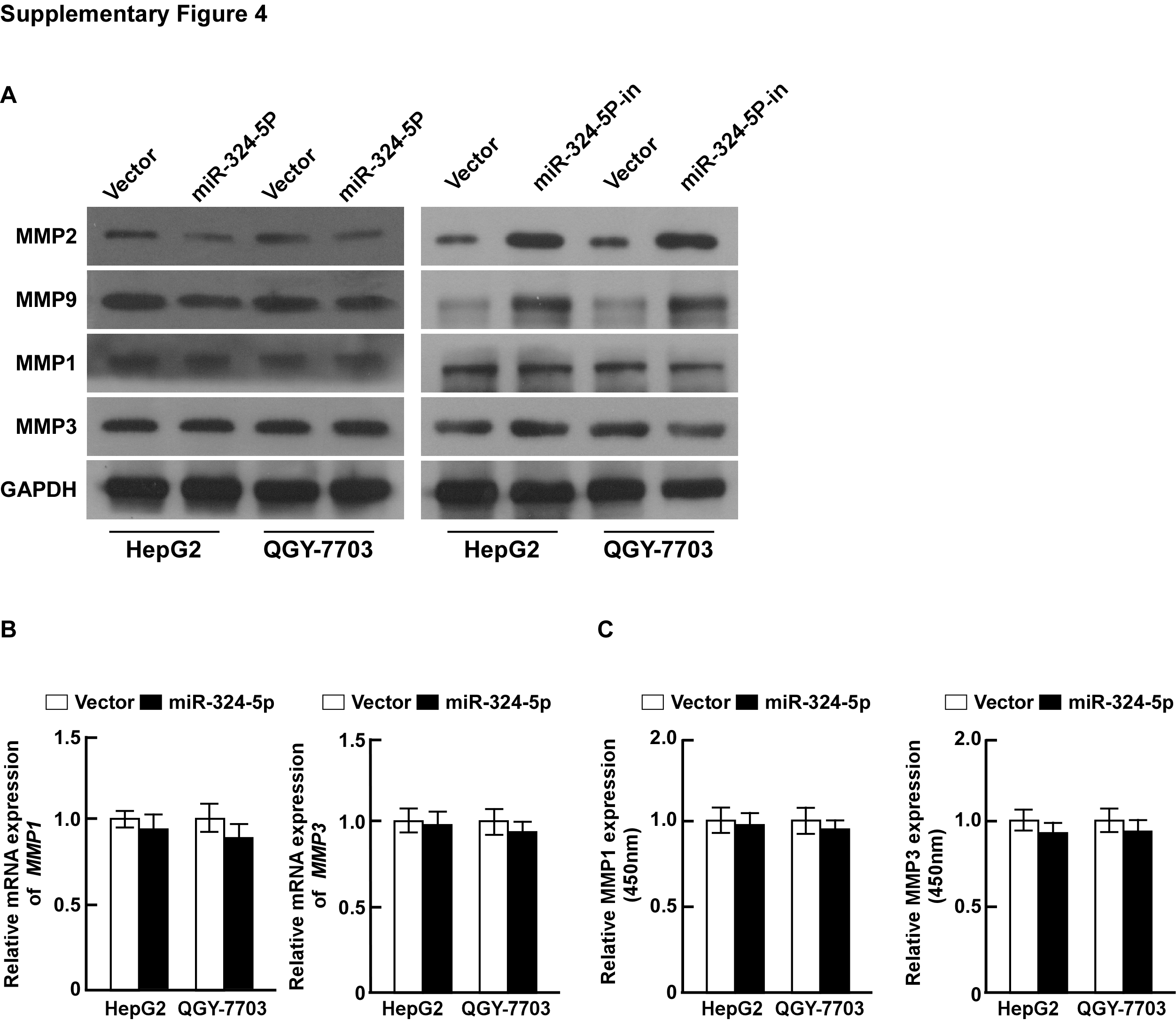

Supplement: S4 Fig — A. The expression levels of MMP1, MMP3, MMP2 and MMP9 protein in HCC cells overexpressing miR-324-5p or transfected with miR-324-5p inhibitor, compared with control cells, by western blotting. GAPDH serves as the loading control. B. The mRNA expression levels of MMP1 and MMP3, determined by Real-time PCR analysis. C. The activity of MMP1 and MMP3 in indicated cells determined by ELISA assay. Each bar represents the mean ± SD of three independent experiments. (TIF) [file pone.0133074.s004.tif]
